# The effects of green cardamom supplementation on blood glucose, lipids profile, oxidative stress, sirtuin-1 and irisin in type 2 diabetic patients: a study protocol for a randomized placebo-controlled clinical trial

**DOI:** 10.1186/s12906-017-2068-6

**Published:** 2018-01-17

**Authors:** Mohadeseh Aghasi, Shohreh Ghazi-Zahedi, Fariba Koohdani, Fereydoun Siassi, Ensieh Nasli-Esfahani, Ali Keshavarz, Mostafa Qorbani, Hoorieh Khoshamal, Asma Salari-Moghaddam, Gity Sotoudeh

**Affiliations:** 1 Department of Community Nutrition, School of Nutritional Sciences and Dietetics, Tehran University of Medical Sciences, Tehran, Iran; 2 Department of Cellular and Molecular Nutrition, School of Nutritional Sciences and Dietetics, Tehran University of Medical Sciences, Tehran, Iran; 3 Diabetes Research Center, Endocrinology and Metabolism Clinical Sciences Institute, Tehran University of Medical Sciences, Tehran, Iran; 4 Department of Clinical Nutrition, School of Nutritional Sciences and Dietetics, Tehran University of Medical Sciences, Tehran, Iran; 5 Department of Community Medicine, School of Medicine, Alborz University of Medical Sciences, Karaj, Iran

**Keywords:** Trial protocol, Diabetes, Green cardamom

## Abstract

**Background:**

It has been suggested that the antioxidant, anti-inflammatory and hypolipidemic activities of cardamom may improve diabetes. However, the effect of this spice has not been investigated in diabetic subjects. This study was planned to determine the effects of green cardamom on blood glucose, lipids and oxidative stress status in type 2 diabetic patients.

**Methods/design:**

Eighty overweight or obese patients with type 2 diabetes will be selected. They will be randomly assigned to receive 3 g/d green cardamom or placebo for 10 weeks. The socio demographic, physical activity and 24-h food recall questionnaires will be collected for each subject. Weight, height and waist circumference will be measured. Determination of blood glucose, lipid profile, and oxidative stress biomarkers including serum levels of total antioxidant capacity (TAC), malondialdehyde (MDA), and glutathione peroxidase (GPx) and superoxide dismutase (SOD) in red blood cells will be performed. The homeostasis model assessment-estimated insulin resistance **(**HOMA**-**IR**)** index and the quantitative insulin-sensitivity check index (QUICKI) will be calculated. Also, serum levels of irisin, and Sirtuin1 (SIRT1) will be measured.

**Discussion:**

This trial will be the first study to explore the effects of green cardamom supplementation on glycemic control, lipid profile and oxidative stress in patients with type 2 diabetes mellitus. The results from this trial will provide evidence on the efficacy of green cardamom in type 2 diabetes mellitus.

**Trial registration number:**

(http://www.irct.ir, identifier: IRCT2016042717254N5), Registration date: 23.11.2016.

## Background

Diabetes Mellitus (DM) is a non-communicable disease that affects many persons annually. In 2000, 171 million people suffered fromDM globally, and it has been estimated to reach 366 million people in 2030 [1]. DM increases the risk of cardiovascular disease (CVD) by 2 to 4-fold. Dyslipidemia plays an important role in the pathogenesis of CVD in type 2 diabetes mellitus (T2DM) patients [2].

Studies have shown that high glucose level by stimulating reactive oxygen species (ROS) production damages β-cells and leads to impaired insulin release and insulin resistance [3, 4]. The antioxidant defence system is responsible for the neutralization of ROS; however, β-cells have a poor antioxidant defence system. Therefore, antioxidant supplementation by elevating antioxidant defence capacity can protect against β-cells dysfunction [5].

Many investigations have been executed regarding the relationship between sirtuin-1 (SIRT1) and irisin with the occurrence of DM. SIRT1 is a class III protein deacetylase that is associated with aging, inflammation and CVD [6]. Moreover, it has been suggested that SIRT1 plays an important role in glucose metabolism by stimulating pancreatic insulin release and insulin signaling pathway [7]. Investigations have stated that activation of SIRT1 leads to deacetylation of nuclear factor (NF)-κB, peroxisome proliferator activated receptor (PPAR)-γ and PPAR-γ coactivator 1α (PGC-1α) which have beneficial effect on glycemic indices, obesity and mitochondrial function [6, 7]. PGC-1α is a transcriptional coactivator that regulates energy balance and expression of fatty acids oxidation genes. In addition, it is involved in the improvement of mitochondrial function, insulin sensitivity and alleviation of oxidative stress. PPAR-γ and NF-κB are transcriptional factors that regulate fat metabolism and inflammation, respectively. Therefore, the inhibition of PPAR-γ and NF-κB activity by SIRT1 deacetylation suppresses fat accumulation and inflammatory processes in the human body [6, 8]. Studies have shown that lower SIRT1 activity is involved in the pathogenesis of DM and insulin resistance. Therefore, interventions that increase SIRT1 activity may be beneficial in improvement of insulin resistance and control of DM [7, 8]. Studies have shown that dietary polyphenols by stimulation of SIRT1 activity result in the suppression of NF-κB and activation of PGC-1α; hence improving insulin resistance and lipid metabolism [6].

Irisin is a recently known exercise-mediated myokine that regulates glucose hemeostasis [9]. Irisin contributes to the browning of white adipose tissue by increasing uncoupling protein-1 (UCP-1) in these cells, which results in the improvement of insulin sensitivity and glucose metabolism. UCP-1 is a key molecule that regulates energy expenditure [10]. Studies have indicated that a lower level of irisin is associated with DM and insulin resistance [9]. The activation of PGC-1α is the main stimulator of irisin release, therefore the elevation of irisin level may be a therapeutic approach in the management of insulin resistance and DM. It has been suggested that flavonoids such as quercetin and resveratrol by activation of PGC-1α- and the SIRT1-related signaling pathway contribute to mitochondrial biogenesis [11]. Mitochondria regulates oxidative metabolism in muscle, hence increase in mitochondrial biogenesis results in higher insulin-related glucose uptake by muscle which subsequently improves insulin sensitivity [12].

Green cardamom (*Elettaria cardamomum*) is a dietary spice (in the ginger family) with nutraceutical effects that has antioxidant, anti-inflammatory and anti-carcinogenic properties [13, 14]. In a recent study, Ahmed et al. indicated that cardamom supplementation by supression of α-amylase and α-glucosidase enzymes has anti-diabetic effects and may regulate glucose metabolism [15]. Cardamom volatile oil is composed of terpenes, esters, flavonoids, 1,8-cineole, alpha-terpinyl acetateand limonene [16]. Previous studies have indicated that 1,8-cineole has antioxidant and lipid-lowering effects [17, 18]. Cardamom is high in polyphenolic compounds such as quercetin, kaempferol, luteolin and pelargonidin which possess antioxidant properties [19]. It has been reported that resveratrol and quercetin can increase the deacetylation effect of SIRT1 by 13-fold and 4.6-fold, respectively [20]. Therefore, it has been hypothesized that cardamom antioxidant and anti-inflammatory activities may improve DM. Kandikattu et al. in an experimental model has shown that cardamom can stimulate in vitro superoxide dismutase (SOD), catalase and glutathione activity [21]. However, in two randomized clinical trials, cardamom (3 g/d) had no significant effect on oxidatve stress biomarkers in pre-diabetic and diabetic patients [22, 23]. To the best of our knowledge, there is no clinical trial that has investigated the effects of cardamom on SIRT1 secretion and subsequently PGC-1α, irisin and insulin sensitivity in diabetic patients. Therefore, this study has designed to investigate the effect of *Elettaria cardamomum* on blood glucose, lipid profile and oxidative stress biomarkers in T2DM patients. To determine the mechanism of cardamom effect on blood glucose and lipid levels, blood irisin, and SIRT1 will be assessed.

## Methods and design

### Study design

**Fig. 1:**
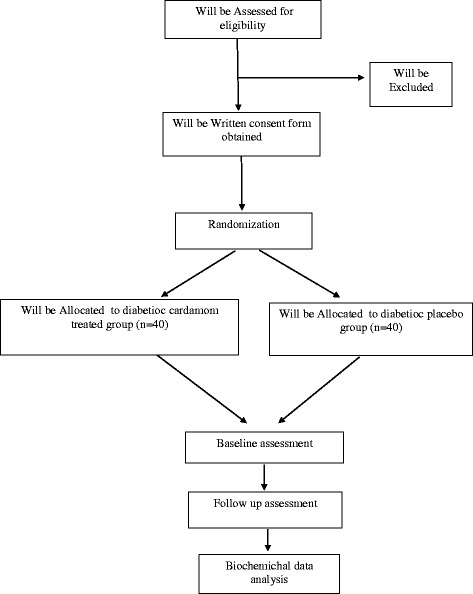
Participants flow diagram

This is a randomized, double blind, clinical trial. The flow chart of the study protocol is presented in Fig. 1. This study was conducted at the Diabetes Research Center, Endocrinology and Metabolism Clinical Sciences Institute, Tehran University of Medical Sciences, Tehran, Iran.

### Sample size:

The sample size was calculated using the following equation:

$$ n=\frac{{\left({Z}_{1-\alpha /2}+{Z}_{1-\beta}\right)}^2\left({S}_1^2+{S}_2^2\right)}{{\left({\mu}_1-{\mu}_2\right)}^2} $$

A total of 40 diabetic patients were calculated in each group (cardamom and placebo groups) based on a significant mean difference of TAC [24], with 95% confidence level, 80% power and additional drop-out rate of 20%.

### Study population

T2DM patients were recruited from the outpatient department of Diabetes Research Center, Endocrinology and Metabolism Clinical Sciences Institute, Tehran University of Medical Sciences, Tehran, Iran. The diagnosis of diabetes will be based on medical records, using the American Diabetic Association (ADA), (fasting blood sugar (FBS) ≥126 mg/dl or 2-h postparndial (2HP) ≥ 200 mg/dl or HbA1c ≥6.5%) [25]. Eligible diabetic patients will be enrolled in the study and written informed consent was obtained at the baseline.

### Inclusion and exclusion criteria

For the present study, T2DM patients, aged 30–60 years, with a diagnosis of T2DM for at least 2 years, HbA1C value greater than 7%, body mass index (BMI) between 25 and 35 kg/m^2^, who will be treated with a stabilized dose of oral anti-diabetic drugs and statins will be included in the study.

Patients will be excluded if: 1) they are pregnant or lactating; 2) have gastrointestinal disorders that interfere with the bowel function, severe hepatic, renal (dialysis), inflammatory and thyroid diseases; 3) being on insulin therapy or need insulin based on expert physician’s opinion 4) have diabetes complications including micro and macrovascular complications; 5) take warfarin, fibrates (PPAR- α ligand), TZD (PPAR- gammaligand) and anti-depressant agents; 6) adhering to a specific diet during the past 3 months; 7) have history of smoking or alcohol intake at least once a week in the past month; 8) receive (at least once a week) herbals, antioxidant, multivitamin/mineral supplements in the past 3 months; 9) they were unstable on the current dose of medications. Patients, who consume less than 90% of their intervention, will also be excluded.

### Randomization

Participants will be randomly allocated into two groups: cardamom and placebo groups and will be followed-up for 10 weeks. Randomizations will be conducted by an assistant using permuted block randomization method and stratified randomization will be used to match participants based on age and gender distribution. The intervention allocation will be blinded for both investigators and participants.

### Intervention

Fruits of *Elettaria Cardamom* will be purchased from Samex Agency (India), by the Traditional Medicine and Research Centre (TMRC), Shahid Beheshti University of Medical Sciences, Tehran, Iran. Cardamom (green cardamom powder) and placebo (rusk powder) capsules will be prepared by TMRC. Each capsule will contain 0.5 g of whole green cardamom powder or rusk powder. Shape, size and color of placebo capsules will be completely similar to the cardamom capsules. All placebo capsules will be placed in the same bag containing the cardamom capsules, in order to have the smell of cardamom.

The voucher number of green cardamom is *E. cardamomum* (L.)Maton, Family: Zingiberaceae, PMP-669. Essential oil, as well as the phenolic and flavonoid contents of whole green cardamom will be measured using gas chromatography–mass spectrometry (GC-MS) and high-performance liquid chromatography (HPLC) at the Institute of Medicinal Plants, Shahid Beheshti University of Medical Sciences. Some of the polyphenolic compounds of green cardamom such as caffeic acid, gallic acid, quercetin and luteolin, which were mentioned in other articles [26, 27] will be determined by HPLC. Cardamom and placebo capsules will be provided to both groups monthly for 10 weeks. Participants will be required to consume 2 capsules (3 g daily) per meal. The last packages of capsules will be checked at the end of the month and the number of remaining capsules will be counted; thereafter, new packages will be delivered to patients. All capsules will be given simultaneously with the usual diabetes care that includes maintenance of the oral anti-diabetic drugs dosage.

Participants will be asked to keep their usual lifestyle including medical nutrition therapy and physical activity level.

### Adherence

To assess patients’ compliance during the 10 weeks, a researcher will be assigned to check them weekly using telephone to discern whether they were consuming the supplements.

### Patient safety

Patients will be monitored weekly during the study period and any occurrence of adverse events will be recorded.

#### Study outcomes

##### Primary outcomes

The primary outcomes of this clinical trial are the changes in HbA1C, FBS, insulin, lipid profile (triglyceride, total cholesterol, HDL-cholesterol, LDL-cholesterol), oxidative stress biomarkers such as TAC, SOD, GPx, MDA, SIRT1 and irisin at the end of the study in comparison with the baseline values.

##### Secondary outcomes

The secondary outcomes of this clinical trial are changes in weight, BMI and waist circumferences (WC).

##### Procedure

After obtaining the informed consent, both groups will be visited at the clinical research center twice: at baseline, and after 10 weeks. Subjects will be interviewed regarding their socio-demographic background at baseline. At each visit, anthropometric indices (weight, height and waist circumference) will be measured, fasting blood sample will be collected and a 24-h food recall and international physical activity questionnaire (IPAQ) will be obtained.

Height will be measured using the SECA stadiometer to the nearest 0.1 cm and weight will be measured using a SECA electronic scale to the nearest 0.1 kg. Waist circumference will be measured midway between the lowest rib and the iliac crest using a non-stretchable measuring tape to the nearest 0.1 kg. BMI will be calculated as weight (kg) divided by height squared (m^2^). Venous blood sample (10 ml) will be drawn after 12-h overnight fasting by trained nurses in seated position to measure biochemical markers. Then blood samples will be centrifuged at 3000 rpm for 10 min at 4 °C to obtain the serum and plasma and will be stored at −80 °C until biochemical analyses. Lipid profiles (TC, HDL, LDL, TG) and glucose level will bemeasured by the enzymatic colorimetric method using kits. Serum insulin will be measured using enzyme-linked immunosorbent assay (ELISA) kit. Insulin sensitivity and insulin resistance will be determined using the QUICKI and HOMA-IR equation, respectively.

$$ \mathrm{QUICK}=1\kern0.5em /\kern0.5em \left(\log \left(\mathrm{fasting}\kern0.5em \mathrm{insulin}\ \upmu \mathrm{U}/\mathrm{ml}\right)\kern0.5em +\kern0.5em \log\ \left(\mathrm{fasting}\  \mathrm{glucose}\ \mathrm{mg}/\mathrm{dl}\right)\right) $$

$$ \mathrm{HOMA}\hbox{-} \mathrm{IR}=\kern0.5em \left(\mathrm{FPI}\left(\mathrm{mU}/\mathrm{l}\right)\kern0.5em \times \kern0.5em \mathrm{FPG}\kern0.5em \left(\mathrm{mmol}/\mathrm{l}\right)\right)/22.5 $$

Serum TAC [28] and level of SOD and GPx in red blood cells will be measured by spectrophotometric methods using kits. Serum irisin and SIRT1 will be measured using ELISA kits.

##### Assessment of dietary intake

To assess participants’ dietary intake, a 24-h food recall will be collected 3 times during the study (at baseline, middle and end of study). Patients will complete food descriptions including food and drinks (brand names), food preparation (ingredients) in detail as much as possible in the last day. Pictures of food commonly consumed in Iran, together with a set of common household measurement tools (glass, cup, soup bowls, plates, teaspoon and tablespoon) will be provided to assist subjects in estimating the portion sizes of the food. Dietary intake will be analysed with Nutritionist version 4.

##### Assessment of physical activity levels

IPAQ will be applied to assess the physical activity level of participants [29]. The IPAQ form comprises walking, moderate-intensity and vigorous-intensity activity and will be expressed as metabolic equivalents per minute (MET-min) per week. The levels of physical activity will be categorized into low, moderate and high, based on the IPAQ criteria.

##### Statistical analysis

All statistical analyses will be performed using SPSS version 21 (SPSS Inc., Chicago, USA). Data will be expressed as mean ± SD for continuous variables and percentage for non-continuous variables. Normality tests will be assessed through Shapiro-Wilk tests carried out on each parameter before analysis. All outcome variables will be assessed based on intention-to-treat and per protocol analysis. Missing data will be entered using “regression imputation” method. To compare the mean differences between the two groups, the independent *t*-test and Mann–Whitney will be used for normal and non-normal data respectively, considering the normality of data. A multivariate repeated-measures regression analysis will be used to assess time effects and time-by-treatment interactions effect on all outcome variables. A *p*-value of less than 0.05 will be considered as significant.

## Ethical considerations

This clinical trial will be conducted after approval by the Ethics Committee of Tehran University of Medical Sciencesand after obtaining informed consent from the participants. Whenever a person is unable to continue supplementation, he/she will be excluded from the study.

## The limitations

One of the limitations of this study is the lack of cooperation of some patients who were replaced with other patients.

## Discussion

T2DM is a common chronic disease worldwide. Recently, herbal medicine has become more popular due to their beneficial effects [30]. Cardamom (*E. cardamomum*) is an expensive spice with an old history [31]. Today, cardamom is grown in India, Thailand, Guatemala, Ceylon and Malay Archipelago [26]. The main characteristics of cardamom include sweet odor, warm nature and mild strong taste that make it a popular spice [31]. Cardamom is rich in antioxidant compounds that cause elevation and activation of the antioxidant defense system [32].

To the best of our knowledge, this is the first randomized controlled trial that will determine the effect of cardamom on glycemic status, lipid profile, oxidative stress biomarkers, SIRT1 and irisin in T2DM. The selection of patients with T2DM is the main strength of the present study. The results of this trial will provide clinical evidence on the effectiveness and safety of cardamom supplementation in patients with T2DM.

## Abbreviations

BMI: Body Mass Index
CVD: Cardiovascular Disease
DM: Diabetes Mellitus
GPx: Glutathione Peroxidase
HOMA**-**IR: Homeostasis Model Assessment-estimated Insulin Resistance
IPAQ: International Physical Activity Questionnaire
MDA: Malondialdehyde
QUICKI: Quantitative Insulin-sensitivity Check Index
ROS: Reactive Oxygen Species
SIRT1: Sirtuin1
SOD: Superoxide Dismutase
T2DM: Type 2 Diabetes Mellitus
TAC: Total Antioxidant Capacity
